# Reduction of Plasma BCAAs following Roux-en-Y Gastric Bypass Surgery Is Primarily Mediated by FGF21

**DOI:** 10.3390/nu15071713

**Published:** 2023-03-31

**Authors:** Harsh Shah, Alyssa Kramer, Caitlyn A. Mullins, Marie Mattern, Ritchel B. Gannaban, R. Leigh Townsend, Shawn R. Campagna, Christopher D. Morrison, Hans-Rudolf Berthoud, Andrew C. Shin

**Affiliations:** 1 Neurobiology of Nutrition Laboratory, Department of Nutritional Sciences, College of Human Sciences, Texas Tech University, Lubbock, TX 79409, USA; 2 Neurobiology of Nutrition & Metabolism Department, Pennington Biomedical Research Center, Louisiana State University System, Baton Rouge, LA 70808, USA; 3 Department of Chemistry, University of Tennessee, Knoxville, TN 37996, USA

**Keywords:** RYGB, branched-chain amino acids, GLP-1, PYY, glycemic control

## Abstract

Type 2 diabetes (T2D) is a challenging health concern worldwide. A lifestyle intervention to treat T2D is difficult to adhere, and the effectiveness of approved medications such as metformin, thiazolidinediones (TZDs), and sulfonylureas are suboptimal. On the other hand, bariatric procedures such as Roux-en-Y gastric bypass (RYGB) are being recognized for their remarkable ability to achieve diabetes remission, although the underlying mechanism is not clear. Recent evidence points to branched-chain amino acids (BCAAs) as a potential contributor to glucose impairment and insulin resistance. RYGB has been shown to effectively lower plasma BCAAs in insulin-resistant or T2D patients that may help improve glycemic control, but the underlying mechanism for BCAA reduction is not understood. Hence, we attempted to explore the mechanism by which RYGB reduces BCAAs. To this end, we randomized diet-induced obese (DIO) mice into three groups that underwent either sham or RYGB surgery or food restriction to match the weight of RYGB mice. We also included regular chow-diet-fed healthy mice as an additional control group. Here, we show that compared to sham surgery, RYGB in DIO mice markedly lowered serum BCAAs most likely by rescuing BCAA breakdown in both liver and white adipose tissues. Importantly, the restored BCAA metabolism following RYGB was independent of caloric intake. Fasting insulin and HOMA-IR were decreased as expected, and serum valine was strongly associated with insulin resistance. While gut hormones such as glucagon-like peptide-1 (GLP-1) and peptide YY (PYY) are postulated to mediate various surgery-induced metabolic benefits, mice lacking these hormonal signals (GLP-1R/Y2R double KO) were still able to effectively lower plasma BCAAs and improve glucose tolerance, similar to mice with intact GLP-1 and PYY signaling. On the other hand, mice deficient in fibroblast growth factor 21 (FGF21), another candidate hormone implicated in enhanced glucoregulatory action following RYGB, failed to decrease plasma BCAAs and normalize hepatic BCAA degradation following surgery. This is the first study using an animal model to successfully recapitulate the RYGB-led reduction of circulating BCAAs observed in humans. Our findings unmasked a critical role of FGF21 in mediating the rescue of BCAA metabolism following surgery. It would be interesting to explore the possibility of whether RYGB-induced improvement in glucose homeostasis is partly through decreased BCAAs.

## 1. Introduction

Diabetes prevalence is surpassing 11% in the United States with over 37 million individuals and is increasing in parallel with that of obesity [1]. Hyperglycemia and insulin resistance associated with Type 2 diabetes (T2D) are major risk factors for cardiovascular, renal, and liver diseases [2,3,4]. Anti-hyperglycemic medications such as metformin, thiazolidinediones (TZDs), and sulfonylureas are often prescribed to patients with T2D to lower their HbA1c and improve glycemic control, but the effect is suboptimal. Less than 20% of patients with T2D are able to reach the treatment targets proposed by the American Diabetes Association [5,6], although therapeutic advances in the last decade by GLP-1 receptor agonists, SGLT2 inhibitors, dual receptor agonists such as tirzepatide [7], as well as a triple combinatorial therapy [8] have begun to provide promising results.

In the last 10 years, bariatric surgery has rapidly risen as an alternative treatment option for T2D, with Roux-en-Y gastric bypass (RYGB) as one of the most popular surgical modalities. In addition to inducing a significant loss of excess body weight and fat mass, RYGB has shown to be very effective in both short- and long-term improvement in glycemic control and insulin sensitivity, often better than those achieved by conventional medical therapy with or without lifestyle interventions [9,10]. Owing to its remarkable beneficial effects on diabetes remission, RYGB is becoming an attractive approach as a “metabolic surgery” to treat and/or cure T2D. However, not only does the procedure come at a high price, but there is also a limited medical capacity for bariatric surgery in the face of an increasing prevalence rate of T2D. A better understanding of the mechanisms by which RYGB surgery markedly improves glucose homeostasis may facilitate the development of noninvasive strategies that can deliver highly efficacious benefits current medications fail to provide.

Several proposed mechanisms for RYGB-induced remission of diabetes include the overstimulated release of incretins [11], alterations in intestinal glucose metabolism [12], changes in bile acid release/signaling [13], and a modified gut microflora [14]. A novel factor that has been brought to the spotlight recently is circulating branched-chain amino acids (BCAAs) and/or their metabolites that are effectively reduced following RYGB surgery in humans [15,16,17,18]. This is physiologically relevant because while BCAAs are essential amino acids we need to obtain from food sources, they are consistently shown to be elevated in individuals with obese/insulin-resistant or T2D [16,19,20,21,22]. More importantly, chronic BCAA supplementation has been shown to impair glucose metabolism and insulin sensitivity [22,23] possibly by hyperactivating mTOR signaling and suppressing mitochondrial capacity [19,22,24,25], while BCAA deprivation or restriction can restore/improve them [26,27,28,29]. However, the potential mechanisms by which RYGB surgery successfully lowers plasma BCAAs and whether they are associated with improved glycemic control that occurs relatively early following surgery is poorly understood.

In this study, we examined BCAA metabolism and glucose homeostasis in our established mouse model of RYGB. Among others, GLP-1, PYY, and FGF21 are considered as major candidate hormones for RYGB-induced improvement in glycemic control and insulin sensitivity. To determine the role of these hormones in BCAA correction following RYGB, mice that lack both GLP-1 and PYY receptors (GLP1R/Y2R double KO) or FGF21 (FGF21 KO) underwent either a sham or RYGB surgery and their BCAA and glucose metabolism were assessed.

## 2. Materials and Methods

### 2.1. Animals and Diets

The current study focused on examining how RYGB surgery affects BCAA metabolism by using the tissue and sample bank from our previously published studies. Diet-induced obese male C57BL/6J mice (~4–5 months old) were purchased from the Jackson Laboratory and were given access to both regular chow diet (58% carb; 28.5% protein; 13.5% fat; 3.36 kcal/g; Purina 5001, LabDiet, St. Louis, MO, USA) and high-fat diet (20% carb; 20% protein, 60% fat; 5.24 kcal/g; D12492, Research Diets, Inc., New Brunswick, NJ, USA) for 14 weeks [30]. Two-month-old FGF21 KO and wildtype (WT) male mice with a C57BL6/J background were bred at Pennington Biomedical Research Center (PBRC) as described previously [31]. They were rendered obese on the same regular chow (13.5% fat) and high-fat diet (60% fat) for 10 weeks before treatments started. Mice with both GLP-1 and PYY receptor deletion (GLP-1R/Y2R double KO) were generated as described earlier [32]. Male GLP-1R/Y2R KO mice and age-matched WTs were fed a high-fat diet (60% fat) until 14 weeks of age at the Jackson Laboratory, after which they were delivered to PBRC and placed on a two-choice diet (regular chow and high-fat diet) for 4 additional weeks before the experiment began. All mice were housed in the animal facility room with a temperature of 22 ± 2 °C and a 12:12 h light/dark cycle unless otherwise stated below. All studies were conducted in accordance with the National Institutes of Health’s *Guide for the Care and Use of Laboratory Animals,* and the protocol was approved by the Institutional Animal Care and Use Committee (IACUC) at PBRC (PBRC-P-949).

### 2.2. Genotyping

The deletion of GLP-1R and Y2R was confirmed in our earlier study [32]. A quantity of 50 ng of DNA was used to genotype WT mice and FGF21 KO mice using the following primers: forward—ACC CCC TGA GCA TGG TAG A, reverse—GCA GAG GCA AGT GAT TTT GA. We used a touchdown PCR protocol as suggested by the Jackson Laboratory (stock no. 033846). The PCR product was separated on 1% agarose by gel electrophoresis and visualized using an SYBR Safe DNA gel stain.

### 2.3. Treatments

Animals were divided into three groups and underwent either sham or RYGB surgery or caloric restriction (given 50–70% of presurgical ad libitum intake) to match their body weight to that of the RYGB group. Preweighed food was provided between 10:00 a.m.–12:00 p.m. each day. Lean C57Bl6/J mice on regular chow diet were added as an additional control group for the first RYGB study described here. For the FGF21 KO and GLP-1R/Y2R KO studies, both WT and KO mice were subjected to sham or RYGB surgery or caloric restriction (WM). Bariatric surgical procedures were performed as described previously [33]. Sham surgery involved a laparotomy only without transections, while RYGB surgery involving intestinal resections and anastomosis resulted in the creation of a biliopancreatic limb (7–9 cm), common limb (18–20 cm), and a Roux limb (5–6 cm) that was attached to a small gastric pouch. All animals were single-housed postsurgically and were provided with solid regular chow and high-fat diet from Day 1.

### 2.4. Body Weight and Food Intake

In the first RYGB study with normal C57Bl6/J mice, and in the FGF21 KO study, body weight and caloric intake were measured every day throughout the treatment period. In the GLP-1R/Y2R double KO study, the body weight of mice after sham or RYGB surgery were measured every 2–3 days while that of WM group was measured every day. Daily caloric intake (kcal) combined from regular chow and high-fat intake was monitored for 10 days postoperatively. An accurate measurement of food intake was made possible by housing mice individually in a cage with a wire mesh floor to collect any spillages.

### 2.5. Glucose Tolerance Test (GTT)

In the GLP-1R/Y2R KO study, 4 h fasted mice underwent an intraperitoneal glucose tolerance test (ipGTT; 1.5 g/kg BW with 30% D-glucose) at 4 weeks postsurgery. Blood glucose from the tail vein was monitored before and after glucose injection (*t* = 0, 15, 30, 60, 120 min) by a hand-held glucometer (Onetouch Ultra Glucometer, LifeScan INC, Milpitas, CA, USA). In FGF21 KO study, ipGTT (2 g/kg BW) was performed on mice 6 weeks after surgery. Mice were fasted overnight before undergoing GTT. The area under the curve (AUC) from each group’s baseline was calculating using the trapezoidal method.

### 2.6. Tissue and Blood Collection

Mice in the first RYGB study, GLP-1R/Y2R KO study, and FGF21 KO study were euthanized by decapitation at 2 weeks, 16 weeks, and 11 weeks post-surgery, respectively. All mice were fasted for 3–4 h before sacrifice in the morning. Trunk blood was collected in EDTA-coated tubes. For FGF21 WT and KO mice, blood from the tail vein was collected in heparin-treated tubes before GTT at 6 weeks postsurgery. Brains and peripheral organs including liver and white adipose tissues were harvested, snap-frozen in liquid nitrogen, and stored at −80 °C prior to processing. Collected blood was immediately treated with heparin/EDTA, a protease inhibitor (Sigma, St. Louis, MO, USA), a DDP-IV inhibitor (EMD Millipore, St. Charles, MO, USA), and Pefabloc SC (Roche, Indianapolis, IN, USA). Plasma was separated through centrifugation at 4 °C and stored at −80 °C for insulin and BCAA analyses.

### 2.7. Fasting Blood Glucose, Plasma Insulin, and HOMA-IR

The fasting blood glucose of mice from the first RYGB study was measured from trunk blood during sacrifice at 2 weeks postsurgery, while it was measured at 16 weeks postsurgery for mice in the GLP-1R/Y2R KO study. FGF21 WT and KO mice had their blood glucose measured from the tail vein after overnight fasting at 6 weeks after surgery. Following the collection of trunk blood and centrifugation, plasma insulin was measured by a Milliplex assay (EMD Millipore, St. Charles, MO, USA). HOMA-IR was calculated from the fasting values of glucose and insulin.

### 2.8. BCAA Assay

The total concentration of BCAAs (leucine, isoleucine, and valine) from plasma samples were measured as previously described by Beckett [34]. Briefly, L-Leucine, L-valine, and L-isoleucine were converted by the leucine dehydrogenase in the presence of excess NAD+ into their corresponding α-keto products plus ammonia and NADH. The generated NADH was then detected as absorbance via a spectrophotometer. Sample absorbance values were used to calculate BCAA concentrations in µM based on the linear standard curve equation.

### 2.9. Metabolomics

For the serum amino acid analysis in the first RYGB study, targeted metabolomics was performed by the Biological and Small Molecule Mass Spectrometry Core at the University of Tennessee as previously described [35]. Briefly, the extraction solvent consisting of 40:40:20 HPLC-grade methanol, acetonitrile, and water with 0.1 M formic acid was added to the sample-containing 1.5 mL tubes. Following extraction for 20 min at −20 °C, samples were centrifuged for 5 min and supernatants were transferred to new vials. Pellets were resuspended two more times in 50 μL of extraction solvent, vortexed, and centrifuged to obtain supernatants. The combined supernatants, after drying in nitrogen gas and resuspension in 300 μL of sterile water, were injected in an Ultimate 3000 RS autosampler (Dionex, Sunnyvale, CA, USA) at 4 °C. Samples were analyzed using ultraperformance liquid chromatography (UPLC)/high-resolution MS in negative ionization mode, and metabolites were identified based on exact masses (±5 ppm) and retention times (±1 min) from a standard library using the open source software package, Metabolomics Analysis and Visualization Engine (MAVEN) [36,37]. The area under the curve (AUC) was calculated by integrating the peaks, and the resulting intensities were normalized to tissue mass to obtain metabolite concentrations.

### 2.10. Western Blots

Liver and epididymal white adipose tissues were homogenized in radioimmune precipitation assay (RIPA) buffer (Cell Signaling, cat. #9806) with added protease inhibitor (Roche, cat. #04693132001) in bead tissue lyser (Tissue Lyser LT, cat. #85600) for 5–7 min with an oscillation frequency of 50 Hz. The homogenized mixture was centrifuged in a two-step process to extract the protein (5 min at 2500× *g* and 15 min at 13,000× *g*). Protein quantification was done by a bicinchoninic acid (BCA) protein assay (Thermo Fisher, cat. #23225) and 30 μg of protein extracts was used to separate proteins by using sodium dodecyl sulfate–polyacrylamide gel electrophoresis (SDS-PAGE) and then transferred on to a polyvinylidene difluoride (PVDF) membrane using Trans-Blot^®^ Turbo™ Transfer System, blocked by 5% nonfat milk in Tris-buffered saline with 1% Tween 20 (TBST) for an hour followed by overnight incubation with a primary antibody BCKDHA (Abcam, cat. #ab138460; lot #GR172773-50), Phospho-BCKDHA (Abcam, cat. #ab200577; lot #GR3369441-2), Vinculin (Cell Signaling, cat. #13901S; lot #7), and PEPCK (Cell Signaling, cat. # 6924; lot # 2). The membrane was washed with TBST and was immunoblotted with antirabbit secondary antibody conjugated with horseradish peroxidase (Cell signaling, cat. #7074; lot #31; 1:3000 dilution) for 1 h. After washing the membrane with TBST, Clarity Western ECL (Bio-Rad, cat. #170–5061) was used as substrate reagent and chemiluminescence was captured using ChemiDoc Imaging System. Captured protein bands were quantified by ImageJ software.

### 2.11. RT-qPCR

Liver tissues from the first RYGB study were harvested, frozen immediately, and stored at −80 °C. Total RNA was extracted and purified using RNeasy Plus Universal Mini Kit (Qiagen, Germantown, MD, USA) and the quality and quantity of RNA were measured using a CYTATION 3 imaging reader (BioTek, Winooski, VT, USA). A quantity of 1 µg total RNA was used to prepare cDNA using iScript™ cDNA Synthesis Kit (Biorad, Hercules, CA, USA) with the following reaction protocol in a thermal cycler (Mastercycler, Eppendorf, Hauppuage, NY, USA): 25 °C for 5 min, 46 °C for 20 min, 95 °C for 1 min. All primers were designed using OligoArchitect primer design software and synthesized by Sigma Aldrich (St. Louis, MO, USA). cDNA, at a final concentration of 5 ng/µL, was amplified using SsoAdvanced Universal SYBR Green Supermix (Biorad, Hercules, CA, USA) on a CFX-Connect real-time PCR instrument (Biorad, Hercules, CA, USA). The amplification protocol for all genes was as follows: initial denaturing at 95 °C for 2 min followed by 40 cycles of denaturation at 95 °C for 5 s, annealing at 60 °C for 30 s, and completed with 95 °C for 5 s, 65 °C for 5 s, and 95 °C for 5 s. Ct values were determined using the regression fit module from CFX-Connect and a final relative quantification was done using the ∆∆Ct method with B2M as the reference gene. Genes involved in BCAA catabolism (BCKDH Kinase, BCKDH, BCAT2, PPM1K) were evaluated by RT-qPCR using primers given in Table 1.

### 2.12. Statistical Analysis

Plasma BCAAs, aromatic amino acids (AAAs), fold change in plasma amino acids and hepatic and adipose tissue protein expression of BCKDH, pBCKDH, the pBCKDH/BCKDH ratio (inactivity index), PEPCK, mRNAs, and blood glucose, plasma insulin, and HOMA-IR were analyzed by a one-way ANOVA followed by Tukey’s post hoc test. Body weight, caloric intake, glucose excursion during GTT, and the area under the curve (AUC) were analyzed by a two-way repeated measures ANOVA (time as within-subject factor; treatment as between-subject factor) followed by a Bonferroni post hoc test. A regression analysis was performed with plasma valine as an independent variable and HOMA-IR as a dependent variable. Plasma BCAAs could not be measured from all mice included in the original studies [30,31,32] due to either a lack of samples, an insufficient number of samples, or hemolysis. Therefore, only the animals from which we were able to measure plasma BCAAs were included in this current study, and the data related to energy balance and glycemic control in this subset of mice from the original studies were reanalyzed to accurately examine their associations with BCAA metabolism. All data are presented as mean ± SEM with *p* < 0.05 set as statistically significant.

## 3. Results

### 3.1. Body Weight and Caloric Intake after RYGB Surgery

All mice were placed on diets containing both regular chow and high-fat (HF) food to induce obesity and were continued on those diets postsurgically. Body weight was identical between groups during the pretreatment period (Day −2 and 0; Supplemental Figure S1A). As shown previously [30], mice after RYGB substantially decreased their weight by day 8 and sustained the weight loss until the end of the study. Mice that were calorie-restricted (WM) to match their weight to that of the RYGB group displayed the same weight reduction as expected. Sham surgery led to an initial lowering of body weight and caloric intake mainly due to surgery-induced trauma, but they were quickly recovered to presurgical levels (Supplemental Figure S1B). RYGB surgery resulted in a further drop in food intake initially, but the mice similarly recovered their feeding so that there was no difference in food consumption compared to the sham group after day 7.

### 3.2. RYGB Surgery Corrects BCAA Dysregulation

**Figure 1:**
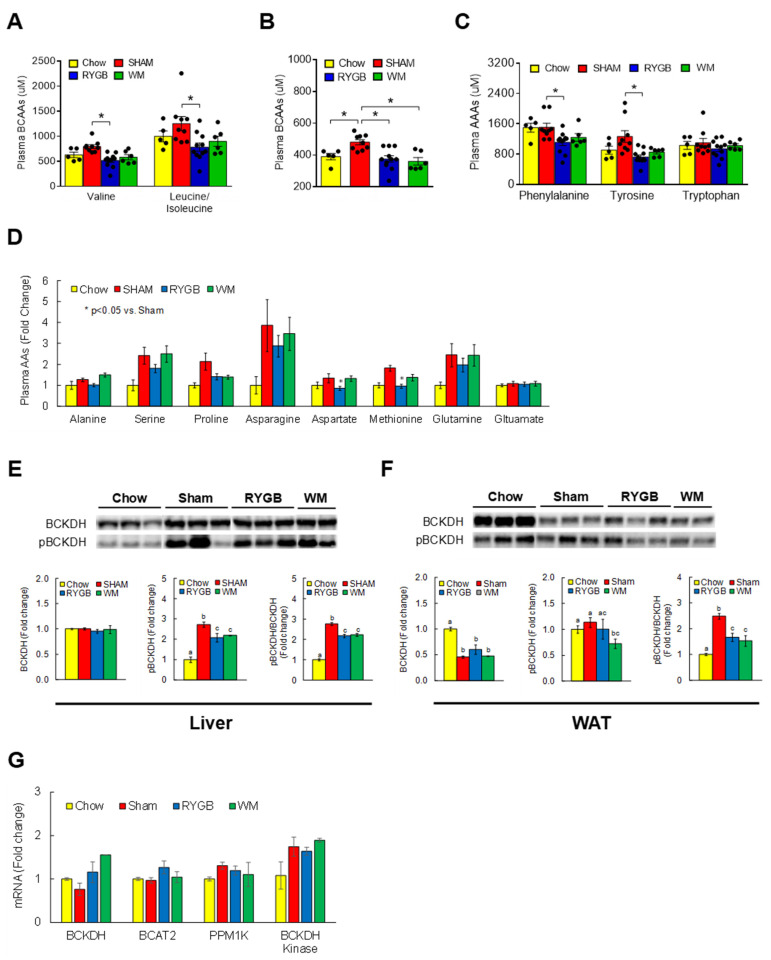
RYGB surgery effectively lowers circulating BCAAs that is independent of food intake. Serum amino acids collected at sacrifice were analyzed by targeted metabolomics via LC-MS. (**A**) Individual serum BCAAs. (**B**) Whole BCAA levels as measured by a separate enzymatic assay. (**C**) Aromatic amino acids (AAAs) and (**D**) other essential and nonessential amino acids via LC-MS. (**E**) Protein expression of BCKDH, the rate-limiting enzyme in the BCAA catabolic pathway and the phosphorylated, inactive state of BCKDH, was measured by Western blots in the liver and (**F**) in epididymal white adipose tissue (WAT). The ratio of pBCKDH/BCKDH indicates the inactivity index; *n* = 5/group. (**G**) mRNA abundance of genes related to BCAA breakdown in the liver as assessed by RT-qPCR. Phosphatase—BCKDH phosphatase; Kinase—BCKDH kinase. Analyzed by a one-way ANOVA followed by Tukey’s post hoc test. Bars with different letters are significantly different from each other; * *p* < 0.05.

To determine if RYGB lowers circulating BCAA levels in mice similar to what has been observed in humans [15,16,17,18,38], we conducted targeted metabolomics to assess serum amino acids by LC-MS. The HF-fed obese sham group displayed higher individual BCAAs (leucine/isoleucine, valine) compared to the chow-fed controls, but this increase was completely normalized following RYGB surgery (Figure 1A). The WM group showed a similar decrease at least partly due to the limited consumption of nutrients including BCAAs (35% restriction) for weight-matching purpose. An RYGB-induced reversal of serum BCAAs was also validated via an independent enzymatic assay that measured a total amount of BCAAs [34] (Figure 1B). Interestingly, serum levels of phenylalanine and tyrosine, the aromatic amino acids (AAAs) shown to be one of the earliest predictive markers of T2D [39], were also found to be significantly decreased following RYGB surgery (Figure 1C). No difference was observed for other amino acids across treatments with the exception of aspartate and methionine (Figure 1D), indicating a rather specific effect of RYGB surgery on BCAAs and AAAs. Branched-chain α-ketoacid dehydrogenase (BCKDH) is the rate-limiting enzyme for the decarboxylation and oxidation of BCAAs mainly in tissues such as liver and heart [40]. Whereas sham obese mice significantly enhanced phosphorylated, inactive state of hepatic BCKDH, this was partially reversed in RYGB and WM groups as evidenced by a lower inactivity index (ratio of pBCKDH to total BCKDH; Figure 1E). A similar induction in BCKDH activity was also observed in epididymal white adipose tissue (WAT; Figure 1F), which is in keeping with BCAA catabolism in WAT playing an important role in regulating systemic BCAA levels [41]. We did not observe any changes in the mRNA abundance of hepatic genes related to BCAA breakdown (Figure 1G), supporting the notion that the enzymatic activity of BCKDH is primarily regulated by posttranslational covalent modification [42].

### 3.3. RYGB-Induced Restoration of BCAA Metabolism Is Associated with Enhanced Insulin Sensitivity

**Figure 2:**
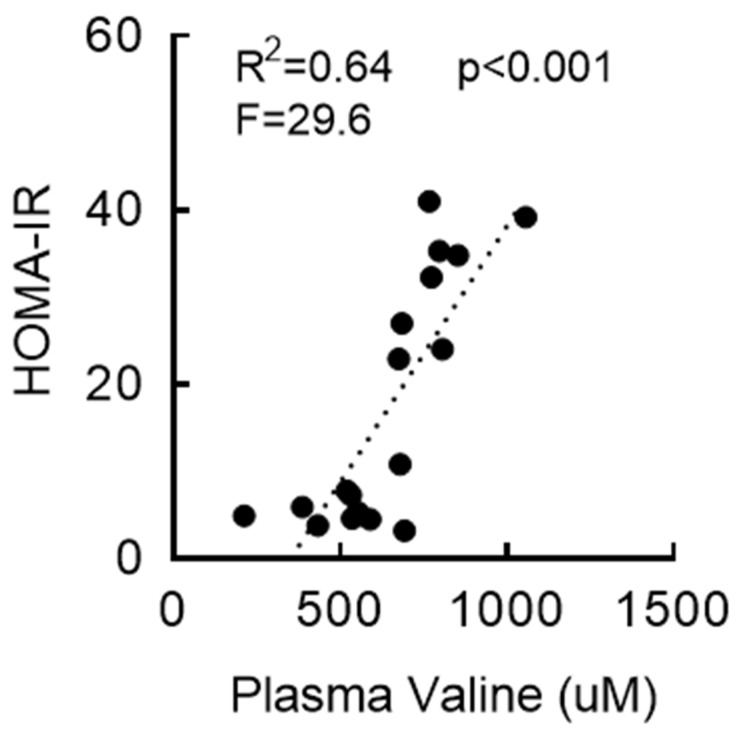
Insulin sensitivity following RYGB is associated with serum valine. Fasting blood parameters were evaluated from the trunk blood collected at the end of sacrifice. Regression analysis with serum valine as an independent variable and HOMA-IR as a dependent variable.

RYGB surgery can improve glycemic control in relatively early years (≤2 years postprocedure) that is often independent of weight loss [15,43,44,45]. While RYGB mice did not show any change in fasting blood glucose compared to sham mice (Supplemental Figure S2A), fasting serum insulin levels were significantly decreased at two weeks postsurgery (↓83%; Supplemental Figure S2B). HOMA-IR, a surrogate indicator of insulin resistance, was more than 40-fold higher in the sham group compared to that in the nonsurgical chow group, but this was completely normalized in RYGB and WM mice (Supplemental Figure S2C). In addition, a linear regression model indicated a positive, statistically significant relationship between serum valine and HOMA-IR and that 64% of the variance in HOMA-IR was explained by serum valine levels (R^2^ = 0.64, *p* < 0.001, F = 29.6; Figure 2). These findings reveal a strong association between BCAA metabolism and fasting insulin and HOMA index and supports the possibility that reduced circulating BCAAs following RYGB surgery may at least partially contribute to improved insulin sensitivity and/or glucose metabolism.

### 3.4. GLP-1R/Y2R Signaling Is Not Critical for Lowered Plasma BCAAs or Improved Glucose Metabolism following RYGB Surgery

**Figure 3:**
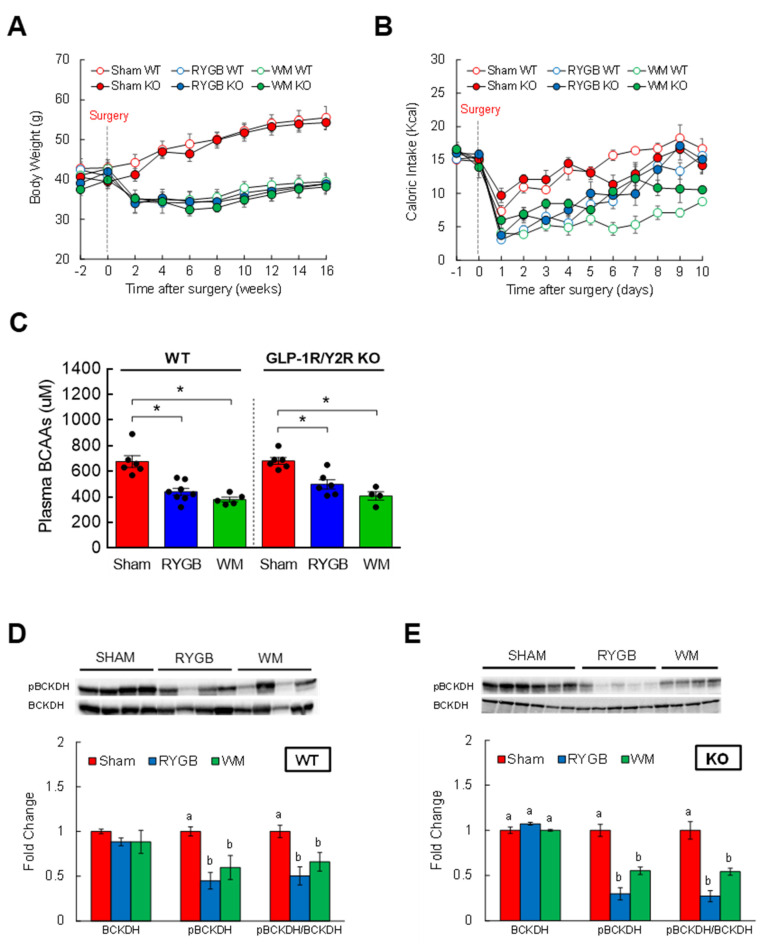
The ability of RYGB to lower plasma BCAAs is intact in mice lacking both GLP-1 and PYY signaling. Six-week-old male WT (*n* = 5–8/group) or GLP-1R/Y2R double KO mice (*n* = 4–6/group) were rendered obese on a 60% high-fat diet for 8 weeks prior to sham or RYGB surgery or caloric restriction. (**A**) Body weight before and after treatments for a 16-week period. (**B**) Daily caloric intake before and after treatments for 10 days. Analyzed by a two-way repeated measures ANOVA followed by a Bonferroni post hoc test. (**C**) Fasting plasma BCAAs measured at 16 weeks postoperatively. (**D**) Protein expression of BCKDH, its phosphorylated state, and inactivity index in WT or (**E**) in GLP-1R/Y2R KO mice. WT: *n* = 5–6/group; KO: *n* = 4–6/group. Analyzed by a one-way ANOVA followed by Tukey’s post hoc test. Bars with different letters are significantly different from each other; * *p* < 0.05.

**Figure 4:**
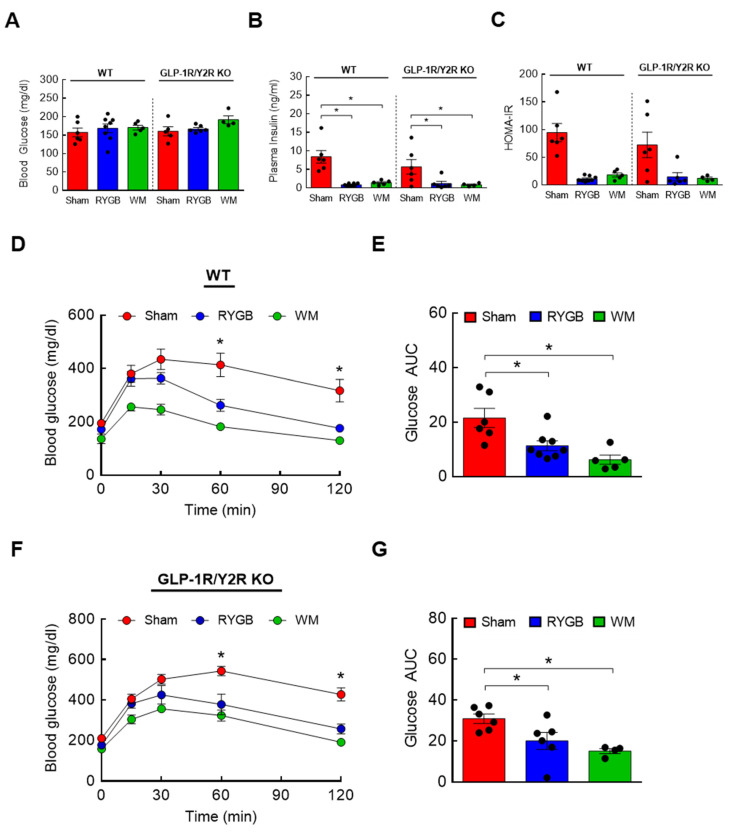
GLP-1/Y2R KO mice are able to improve glucose tolerance and insulin sensitivity in response to RYGB. Six-week-old male WT (*n* = 5–8/group) or GLP-1R/Y2R double KO mice (*n* = 4–6/group) were rendered obese on a 60% high-fat diet for 8 weeks prior to sham or RYGB surgery or caloric restriction. Trunk blood and livers were collected at sacrifice. (**A**) Blood glucose, (**B**) plasma insulin, and (**C**) HOMA-IR. Analyzed by a one-way ANOVA followed by Tukey’s post hoc test. (**D**,**E**) Intraperitoneal glucose tolerance test (GTT; 1.5 g/kg ip) was conducted after a 6 h fasting at 4 weeks postsurgery in WT mice and (**F**,**G**) in GLP-1R/Y2R KO mice. Analyzed by a two-way repeated measures ANOVA followed by a Bonferroni post hoc test. * *p* < 0.05.

Two candidate gut hormones thought to be responsible for surgery-induced improvement in glycemic control are glucagon-like peptide 1 (GLP-1) and peptide YY (PYY) that are released mainly from the small intestine [43,46,47]. Their postprandial rise in circulation is blunted in obesity but hyperstimulated postsurgically, so it is reasonable to hypothesize that a RYGB-induced lowering of plasma BCAAs, one of the culprits that impair glucose metabolism and insulin sensitivity, is in part attributable to enhanced GLP-1 and/or PYY signaling. If this is correct, then abrogating their hormonal actions should abolish the normalization of BCAA metabolism. To test this, mice with global GLP-1R and Y2R deletion (GLP-1R/Y2R double KO) underwent either a sham or RYGB surgery or caloric restriction (WM). Throughout the 16-week postsurgical period, the sham-surgery groups showed a persistent increase in body weight whereas the RYGB groups reduced their body weight by 15–20% regardless of the genotype (Figure 3A). As shown in the first experiment (Supplemental Figure S1), mice after RYGB surgery substantially dropped their caloric intake in the first few days that was fully recovered by day 8 and was no longer different from that of sham mice (Figure 3B). Similar to their body weight data, the lack of GLP-1R and Y2R did not alter their feeding response to RYGB surgery. While these findings revealed a robust effect of RYGB surgery on energy balance that is largely independent of GLP-1 and PYY, it is still possible that the beneficial effects of RYGB surgery on nutrient handling may require their hormonal actions. Interestingly, we found that RYGB surgery led to significantly decreased plasma BCAA levels in not only WTs (uM; 675 ± 46 sham vs. 439 ± 27 RYGB vs. 379 ± 18 WM; *p* < 0.01) but also in GLP-1R/Y2R KO mice (680 ± 27 sham vs. 497 ± 36 RYGB vs. 406 ± 33 WM; *p* < 0.05; Figure 3C). In line with this observation, both WT and KO mice were able to enhance hepatic BCAA breakdown in response to RYGB surgery as evidenced by a significantly decreased inactivity index (↓50–70%; Figure 3D,E). Based on these results, we speculated that RYGB in GLP-1R/Y2R KO mice would similarly improve glucose homeostasis. As expected, both WT and KO mice following surgery exhibited a significantly lower plasma insulin and HOMA-IR without changes in fasting blood glucose (Figure 4A–C). More importantly, RYGB surgery led to a markedly improved glucose homeostasis in both WT (Figure 4D,E) and KO groups (Figure 4F,G) compared to sham surgery, as indicated by a significantly lower blood glucose excursion during glucose tolerance test. Collectively, these results suggest that a combined GLP-1 and PYY signaling does not play a major role in improving BCAA metabolism or glycemic control following RYGB surgery.

### 3.5. FGF21 Is Required for RYGB-Induced Reinstatement of BCAA Metabolism

**Figure 5:**
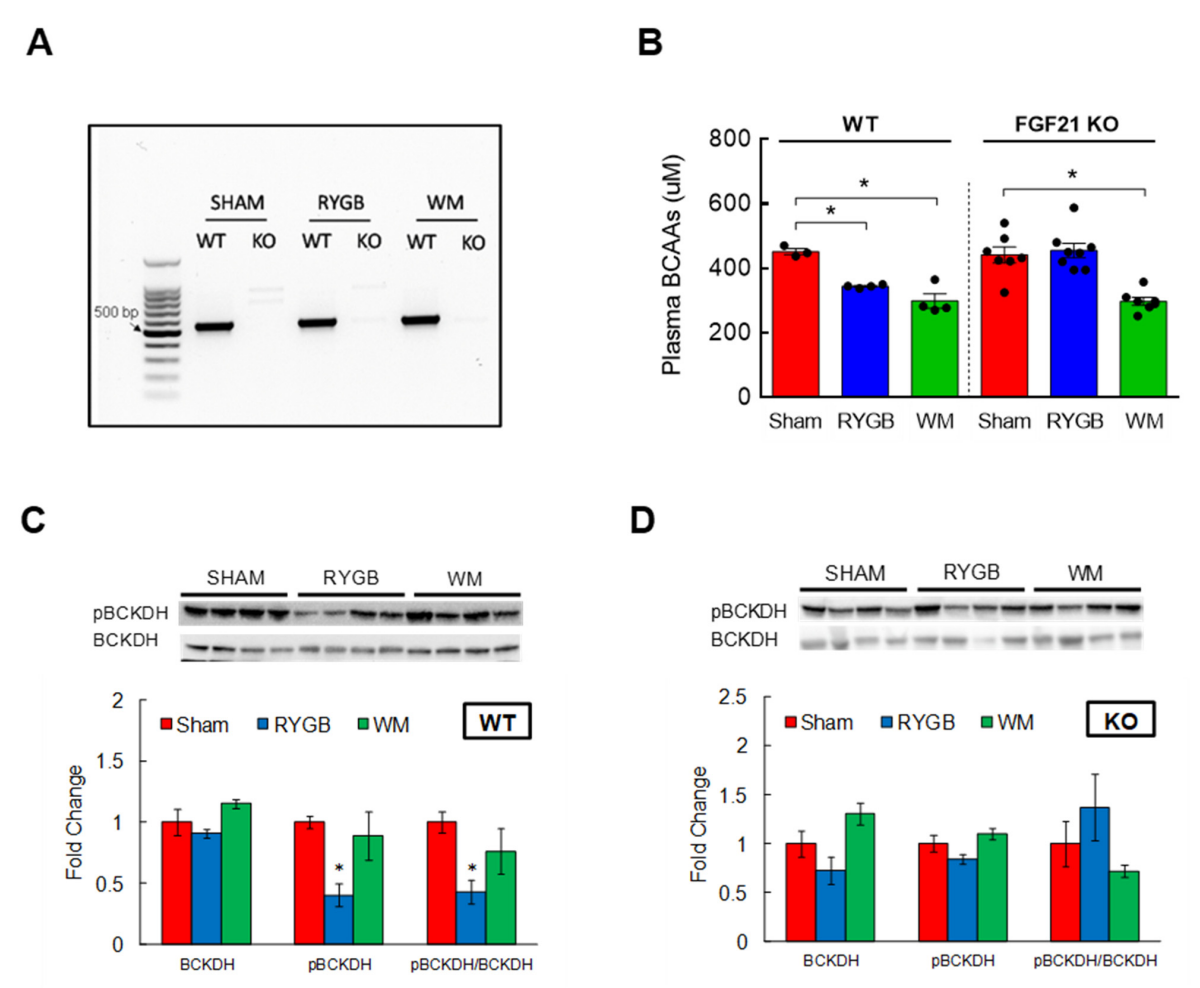
Enhanced BCAA metabolism following RYGB surgery is lost in FGF21 KO mice. Eight-week-old male WT (*n* = 3–4/group) or FGF21 KO mice (*n* = 7–8/group) were fed a two-choice diet (regular chow or 60% high-fat diet) for 10 weeks before being subjected to sham or RYGB surgery or caloric restriction. (**A**) Verification of FGF21 deletion by PCR. (**B**) Plasma BCAAs measured from tail blood after overnight fasting at 6 weeks postsurgery. (**C**) Protein expression of hepatic BCKDH, its phosphorylated state, and inactivity index in WT or (**D**) in FGF21 KO mice after sacrifice at 11 weeks postoperatively. Analyzed by a one-way ANOVA followed by Tukey’s post hoc test. * *p* < 0.05 compared to sham.

Fibroblast growth factor 21 (FGF21) is a hormone primarily expressed in the liver [48]. While prolonged fasting increases the production and release of FGF21 to stimulate gluconeogenesis [49], a number of studies have shown that FGF21 administration in animals with obesity or diabetes effectively improves insulin sensitivity and glucose metabolism [50,51]. Further, bariatric surgery in obese and diabetic state enhances FGF21 sensitivity that in turn promotes glucose homeostasis [52]. Considering the ability of BCAAs to impair glycemic control, we hypothesized that FGF21 would mediate the RYGB-induced reduction in plasma BCAAs to accomplish its glucoregulatory action. To this end, we sought to determine the effects of RYGB surgery on BCAA metabolism in mice with a whole-body FGF21 deletion (FGF21 KO; Figure 5A). In parallel with GLP-1R/Y2R double KO mice, FGF21 KO mice successfully decreased their body weight and maintained weight loss (~25%) in response to RYGB surgery for up to 11 weeks (Supplemental Figure S3A). Caloric intake was also restored after the first week postsurgery and was not different from the intake of WT mice (Supplemental Figure S3B), suggesting that FGF21 signaling is not necessary for RYGB surgery to effectively lower body weight. Interestingly, we obtained completely opposite results while examining BCAA metabolism. Compared to sham surgery, RYGB surgery significantly reduced plasma BCAAs in WT mice as expected (uM; 452 ± 10 sham vs. 344 ± 3 RYGB; *p* < 0.01), but this decrease was clearly abolished in FGF21 KO mice and there was no longer any difference between the sham group and the RYGB group (441 ± 25 sham vs. 455 ± 22 RYGB; Figure 5B). WM mice via caloric restriction lowered plasma BCAAs regardless of FGF21 signaling (299 ± 22 WT; 298 ± 12 KO). While hepatic BCKDH activity was markedly higher in WTs following RYGB surgery (i.e., reduced inactivity index; Figure 5C), in agreement with the failure to lower plasma BCAAs, RYGB surgery in FGF21 KO mice was not able to increase hepatic BCKDH activity to break down and reduce plasma BCAAs (Figure 5D). These results suggest that the ability of RYGB surgery to lower plasma BCAAs requires FGF21 signaling.

### 3.6. The Effects of FGF21 Deletion on Glycemic Control following RYGB Surgery

**Figure 6:**
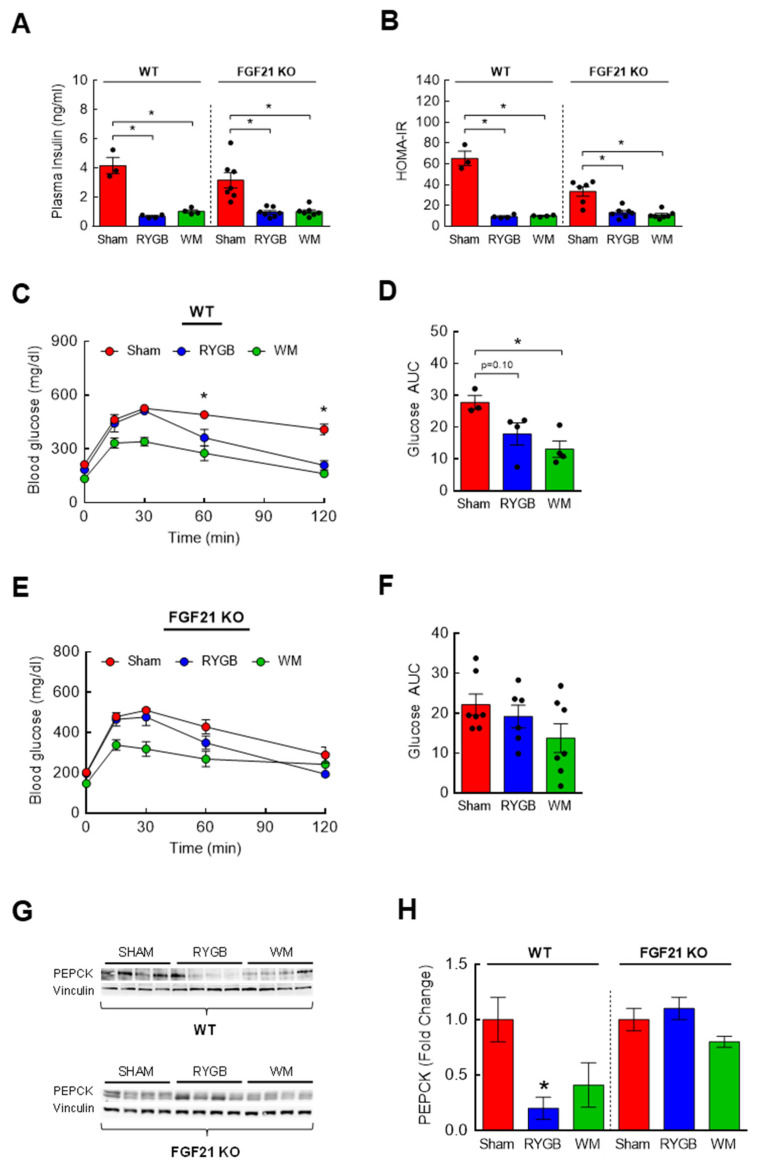
The effects of FGF21 on insulin sensitivity and glycemic control in response to RYGB. Eight-week-old male WT (*n* = 3–4/group) or FGF21 KO mice (*n* = 7–8/group) were fed a two-choice diet (regular chow or 60% high-fat diet) for 10 weeks before being subjected to sham or RYGB surgery or caloric restriction. (**A**) Plasma insulin and (**B**) HOMA-IR calculated from overnight fasted glucose and insulin values at 6 weeks post-surgery. Analyzed by a one-way ANOVA followed by Tukey’s post hoc test. (**C**,**D**) Intraperitoneal glucose tolerance test (GTT; 2 g/kg ip) was conducted after overnight fasting at 6 weeks postsurgery in WT mice and (**E**,**F**) in FGF21 KO mice. Analyzed by a two-way repeated measures ANOVA followed by a Bonferroni post hoc test. (**G**,**H**) Protein expression of hepatic PEPCK in WT and FGF21 KO mice by Western blots. Analyzed by a one-way ANOVA followed by Tukey’s post hoc test. * *p* < 0.05 compared to sham.

BCAA supplementation perturbs while BCAA lowering via dietary restriction or pharmacological intervention improves glucose homeostasis and insulin sensitivity [22,26,28,29,53,54,55,56,57]. Since RYGB surgery is able to both effectively lower plasma BCAAs and restore glycemic control [15,17], the complete lack of BCAA reduction in RYGB-treated FGF21 KO mice implies a possible absence of improved glucose metabolism. In our current study, fasting plasma insulin and HOMA-IR were markedly decreased after RYGB surgery in FGF21 KO mice similar to those observed in WT mice (Figure 6A,B). However, while glucose tolerance was significantly improved in WT RYGB mice compared to WT sham mice at *t* = 60 and 120 min (*p* < 0.05; Figure 6C,D), it appeared impaired after RYGB surgery in FGF21 KO mice (Figure 6E,F) although this could be partly due to the unexpectedly reduced glucose excursion in sham KO mice. Of note, RYGB surgery led to a similar glucose excursion in both WT and FGF21 KO mice. Glycemic control is tightly regulated by the rate of endogenous glucose output and glucose uptake into tissues [58], and bariatric surgery is known to improve glycemic control through suppressing glucose production while stimulating whole-body glucose disposal [59]. Compared to sham surgery, RYGB surgery markedly decreased the rate-limiting enzyme in gluconeogenesis, PEPCK, in the liver of WT mice (↓80%; Figure 6G,H). However, RYGB surgery failed to do so in FGF21 KO mice which is in agreement with the impaired glucose tolerance observed in these mice.

## 4. Discussion

Beyond their elevated levels found in obesity and T2D, it is becoming increasingly clear that BCAAs may exert detrimental effects on glycemic control and insulin action [22,23,26,27,28,29,57]. The effective lowering of plasma BCAAs and their metabolites by bariatric surgeries such as RYGB may potentially contribute to a restored glucose metabolism and remission of diabetes as often observed postsurgically, although the underlying mechanisms have not been explored. Using our established mouse model for RYGB, we demonstrated for the first time a significant reduction of circulating BCAAs following the procedure in diet-induced obese mice as shown in humans, and that this effect was lost in mice that lack the signaling of FGF21 but not of other candidate hormones including GLP-1 and PYY. Our results further revealed that a surgery-induced BCAA reduction was coupled with an improved glucose homeostasis.

While our RYGB mouse model was shown earlier to recapitulate major surgery-induced benefits in humans, including sustained weight/fat loss and improved glycemic control and insulin sensitivity [30,33], the effects of surgery on BCAA metabolism remained unknown. In the current study, a complete normalization of serum BCAA levels following RYGB surgery through two independent tools—a metabolomics platform and an enzymatic assay—validated our mouse model for studying the underlying mechanisms for a restored BCAA metabolism. Moreover, the same observation from a total of three cohorts of mice at 2, 6, or 16 weeks postsurgery clearly suggested that the BCAA correction occurred early on and persisted for a long time. In addition to diet-induced obese mice with sham surgery, the inclusion of chow-fed lean mice and those weight-matched (WM) to RYGB mice by caloric restriction allowed us to better understand how RYGB successfully lowered circulating BCAAs. In line with evidence supporting a clear association between elevated plasma BCAAs and obesity [20,22], a significant reduction of BCAAs in the RYGB group compared to the sham group would lead one to reason that this is likely due to a marked weight loss following the surgery. Indeed, a BCAA lowering of similar magnitude was also observed in calorie-restricted WM mice. However, it is difficult to define this outcome solely as a weight-dependent effect because the WM group not only lost weight but also consumed 40% less food from both HFD and chow diet than the RYGB group to accomplish the same weight loss, which translated into a significantly less BCAA intake and that could partly explain their lower serum BCAAs. An additional group comprising caloric restriction while maintaining the same BCAA intake as that of the RYGB mice would be necessary to test the effect of pure weight loss on BCAA levels. In human studies, unlike RYGB surgery, the same weight loss achieved by dietary intervention is not able to decrease plasma BCAAs [15,17], thus supporting the concept that RYGB-induced BCAA lowering in our mice may be largely weight-loss-independent. Since circulating BCAA levels are partly determined by the amount of dietary BCAA intake, decreased serum BCAAs may be attributed to the suppression of food intake due to surgery. Interestingly, our results reveal that the RYGB-induced BCAA lowering is largely independent of feeding since food intake in RYGB mice was fully restored 9–10 days after surgery and was not different from that of the sham group, and yet their serum BCAAs were significantly lower. Conversely, their food intake was certainly higher than that of chow-fed lean mice or WM mice while displaying similar levels of serum BCAAs. Apart from dietary BCAA consumption, systemic BCAA levels are primarily determined by their breakdown in tissues that have capacity to catabolize BCAAs [40,60]. Our findings of an increased, efficient BCAA catabolism in both liver and white adipose tissues of RYGB mice in spite of a restored food (and BCAA) intake explain the ability of these animals to successfully suppress circulating BCAAs. The degradation of BCAAs in other tissues such as kidney and heart tissues with a moderate/high activity of BCKDH—the rate-limiting enzyme in BCAA catabolism—is also possible, which in turn may contribute to lower BCAAs in mice with RYGB surgery.

As shown in our earlier study [30], both RYGB surgery and calorie restriction were able to markedly lower plasma insulin and display a sign of restored insulin sensitivity as indicated by HOMA index. These benefits following RYGB, in particular, may be at least partly attributed to the reduction of glucogenic amino acids methionine and aspartate that can affect insulin action, as well as a decrease in phenylalanine and tyrosine, the aromatic amino acids associated with impaired insulin secretion [61] that are the earliest predictors for future risk of T2D [39]. Of note, a linear regression analysis demonstrating that plasma valine could successfully predict HOMA index strongly indicated a potential link between BCAAs and the ability of RYGB to improve insulin sensitivity and/or glucose metabolism. 

Due to a substantially over-stimulated release of PYY and the incretin hormone GLP-1 from the gut following RYGB surgery [43], they have been proposed as one of the most promising candidate hormones that may explain the underlying mechanisms for several metabolic benefits of the surgical procedure including restored glycemic control and insulin sensitivity. While it is then reasonable to speculate that GLP-1 and PYY might elicit their glucoregulatory actions in part by reducing plasma BCAAs, we did not observe any differences in the RYGB-induced BCAA lowering or its catabolism between WT and GLP-1R/Y2R KO mice. Likewise, the lack of hormonal signaling did not affect enhanced glucose tolerance following RYGB surgery. These findings extend our previous results [62] that failed to show any changes in surgery-induced weight loss from GLP-1R/Y2R-deficient mice and suggest that GLP-1 and/or PYY signaling does not appear to be a major determinant of metabolic improvement following RYGB. Alternatively, BCAA reduction after surgery may be at least partly mediated by GLP-1 when combined with another incretin hormone, the glucose-dependent insulinotropic polypeptide (GIP). A recent study by Pirro and colleagues [63] demonstrated that tirzepatide, a dual GIP and GLP-1 receptor agonist that improves glycemic control in T2D patients, was also shown to markedly lower plasma BCAAs. Another study [64] showed that either a tirzepatide or a long-acting GIP receptor agonist could enhance insulin sensitivity by augmenting glucose disposal in the WAT of mice lacking GLP-1R. Interestingly, the effect of these agonists on insulin sensitivity was associated with reduced circulating BCAAs, suggesting that rather than GLP-1, another incretin GIP may be the primary driver that lowers BCAA levels and improves glycemic control. The results on plasma GIP levels following the RYGB procedure are inconsistent [65,66,67] most likely due to the differences in the length of the Roux limb created or the obese/diabetic status of the participants, but its potential BCAA-decreasing role following bariatric surgery is a reasonable possibility.

FGF21 is a hormone or hepatokine shown to enhance glucose homeostasis [68]. The underlying mechanisms are not completely understood, but it is thought to occur in part through improved hepatic insulin sensitivity that increases hepatic glucose uptake while simultaneously decreasing hepatic glucose production [69,70]. Since bariatric surgery promotes insulin sensitivity and responsiveness to FGF21 [52], FGF21 was recently proposed as one of the candidate hormones to explain the RYGB-induced improvement in glucose metabolism, although the underlying mechanisms are unclear. We speculated that a lowering of BCAAs by FGF21 may be the missing piece of the puzzle, and indeed, a plasma BCAA reduction following surgery was completely reversed in FGF21-deficient mice. Interestingly, these mice also seemed to lack enhanced glucose tolerance following RYGB surgery. This is further supported by significantly decreased hepatic PEPCK—the rate-limiting enzyme in gluconeogenesis—after RYGB surgery in WTs that is lost in FGF21 KO mice. With that said, it would be difficult to conclude the role of FGF21 on the RYGB-induced glycemic correction based on GTT data here, because RYGB surgery led to similar glucose excursion in both WT and FGF21 KO mice. FGF21 KO mice with sham surgery unexpectedly displayed a lower glucose excursion compared to WT mice, thus minimizing the change in glucose tolerance following RYGB surgery. It is difficult to explain the large difference in glucose tolerance we observed between WT and KO mice after sham surgery. Of note, variable findings on glucose tolerance between these groups have been reported previously [49,69,71]. The discrepancy between studies may come from the differences in animal age, HFD exposure, and/or the degree of insulin and/or FGF21 resistance at the time of testing. This warrants further investigation.

What would be the mechanism by which FGF21 improves BCAA metabolism following RYGB surgery? Whole-body deletion of FGF21 in our study prevented us from determining the precise site(s) of action. FGF21 binds to all four isoforms of FGF receptors (FGFR-1c, 2c, 3c, 4) [72] and these receptors are widely expressed both in the periphery and the brain [73,74]. Current evidence suggests that its weight loss and glucoregulatory effects are largely centrally mediated [49,75,76,77]. Our group has previously shown that hypothalamic insulin signaling is sufficient and required to lower plasma BCAAs mainly by inducing hepatic BCAA catabolism [78,79]. Earlier findings on improved insulin sensitivity in the hypothalamus after RYGB surgery in rats would suggest that an effective lowering of plasma BCAAs postsurgically may be due to a restored hypothalamic insulin action. This raises an interesting possibility that FGF21 may enhance insulin signaling in the CNS to regulate systemic BCAA metabolism. Alternatively, it is also reasonable to speculate that the RYGB-induced reinstatement of hypothalamic insulin signaling decreases plasma BCAAs through an FGF21 action in the liver. Since the liver is the major organ that synthesizes FGF21 [80], it is possible that FGF21 acts in an autocrine/paracrine manner to promote hepatic BCAA catabolic activity that would lead to lower circulating BCAA levels. Whether or not FGF21 can alter the gene expression or the enzymatic activity of BCKDH remains currently unknown. Conducting RYGB surgery in mice with a periphery- or brain-specific blockade of FGF21 signaling and hepatic knockdown of BCKDH will allow us to determine the site of action and further dissect the role of FGF21 in improving BCAA homeostasis.

## 5. Conclusions

Taken together, our findings demonstrated that the RYGB procedure effectively lowered circulating BCAAs in diet-induced obese mice, largely independent of food intake. We further showed that FGF21 signaling, but not GLP-1 or PYY signaling, primarily mediated the RYGB-induced improvement in BCAA homeostasis. Considering the ability of BCAAs to promote insulin resistance, these results offer a promising possibility that RYGB surgery may restore glycemic control by suppressing BCAA levels.

## Figures and Tables

**Table 1:** Primer sequences used.

Gene Name	Type	Sequence (5′→3′)
BCKDH	Forward	GGATGAGGAACAGGAGAAGG
	Reverse	GGAGAAGAGGAGGCTTGG
BCKDH Kinase	Forward	GACAGGTGGACTTAGATGGA
	Reverse	CAAGAATGAGCAGAGCAGAG
PPM1K	Forward	CCTGCTACTTCTCCACTTCA
	Reverse	GCTCATCAATGCGGTTATCC
BCAT2	Forward	GCAGACCTCCAGATTCAGA
	Reverse	TGTTATTCCACTCCACCATCA
B2M	Forward	GAAGCCGAACATACTGAACTG
	Reverse	CTGAAGGACATATCTGACATCTCT

## Data Availability

The data that support the findings of this study area available from the corresponding author upon reasonable request.

## Supplementary Materials

The following supporting information can be downloaded at: https://www.mdpi.com/article/10.3390/nu15071713/s1, Figure S1: Effect of RYGB on body weight and food intake in C57BL/6 mice, Figure S2: RYGB improves insulin sensitivity in HF-fed obese mice, Figure S3: RYGB surgery leads to a similar weight loss in FGF21 KO mice.
