# Supplementary material for: Reduction of Plasma BCAAs following Roux-en-Y Gastric Bypass Surgery Is Primarily Mediated by FGF21
Source: Nutrients. 2023 Mar 31;15(7):1713. doi: 10.3390/nu15071713 (PMC10096671; doi:10.3390/nu15071713)
Supplement: Supplementary file 1 [file nutrients-15-01713-s001.zip › nutrients-2272987-supplementary.pdf]

Supplemental Figure S1

A

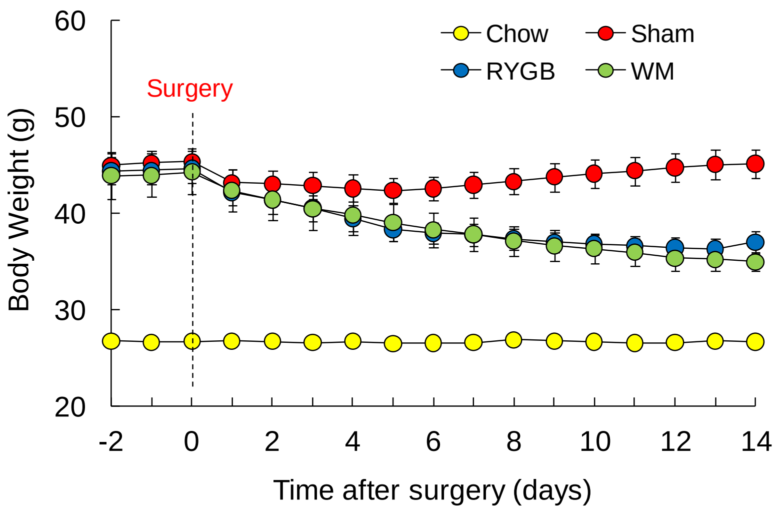

B

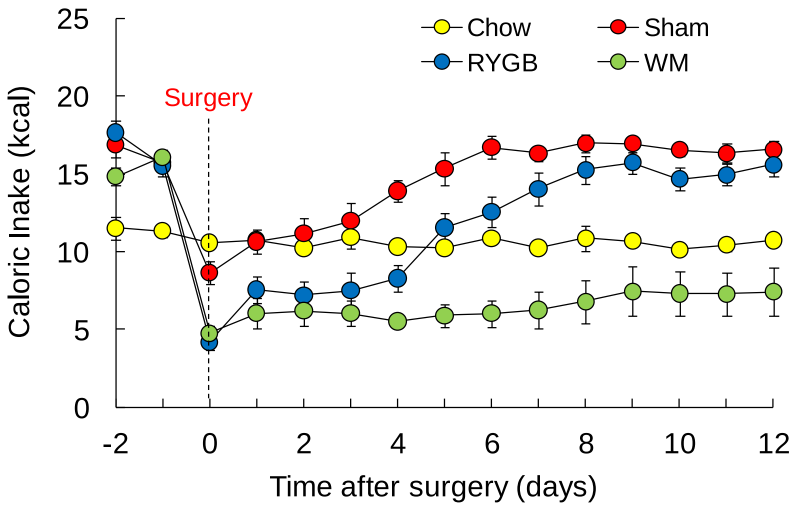

Supplemental Figure S2

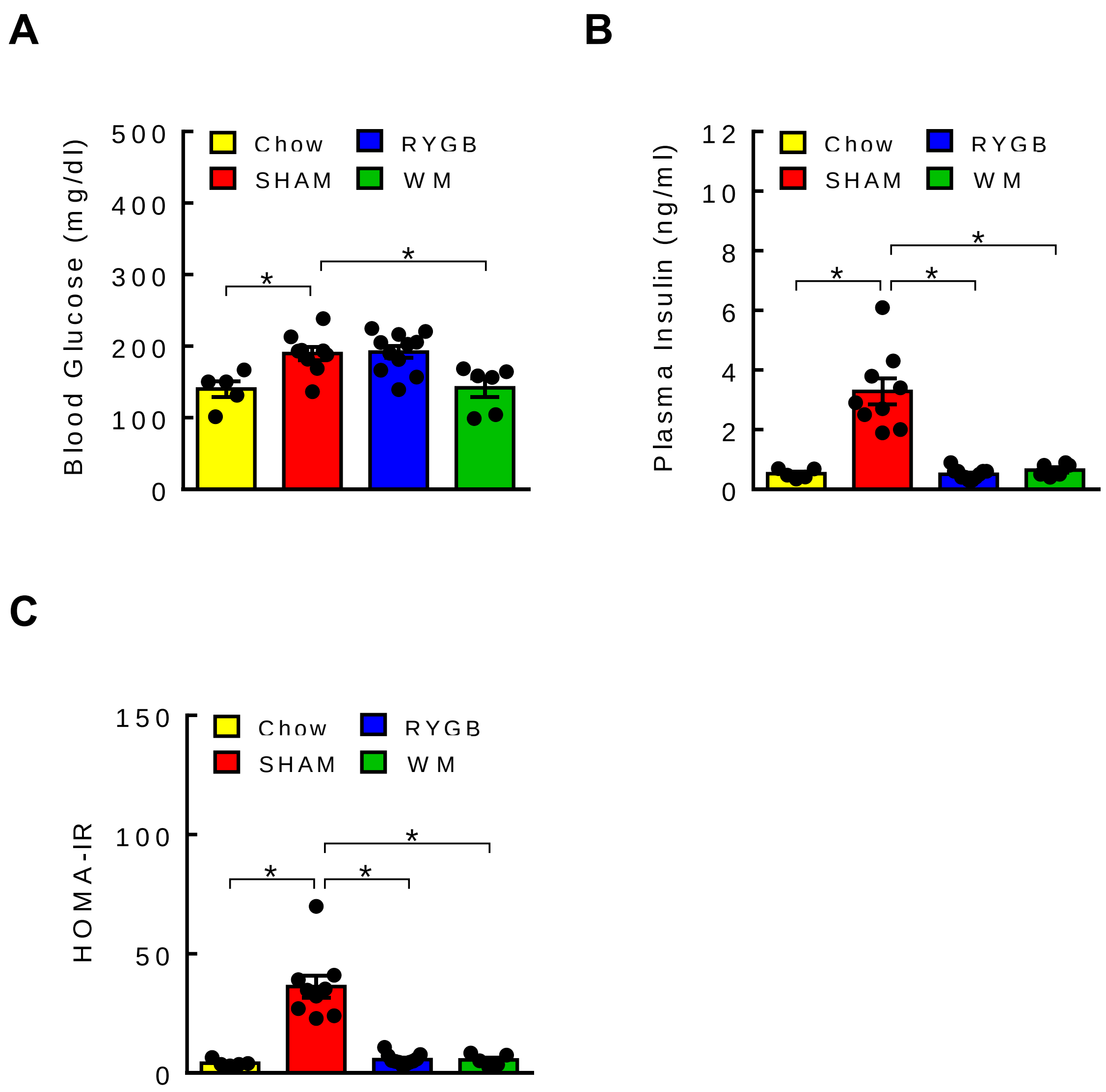

Supplemental Figure S3

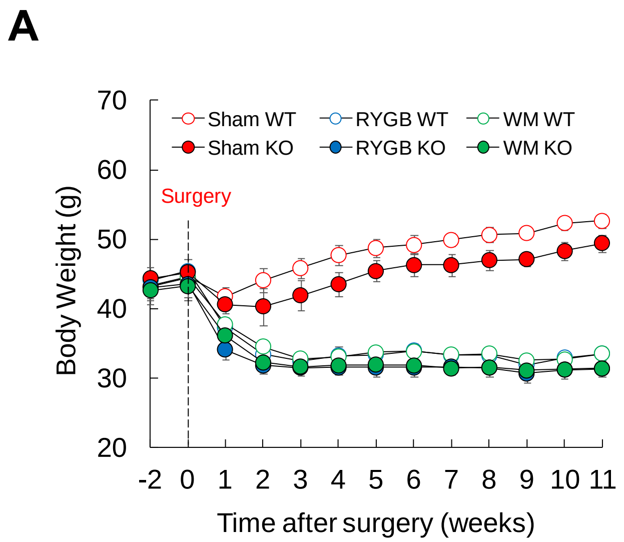

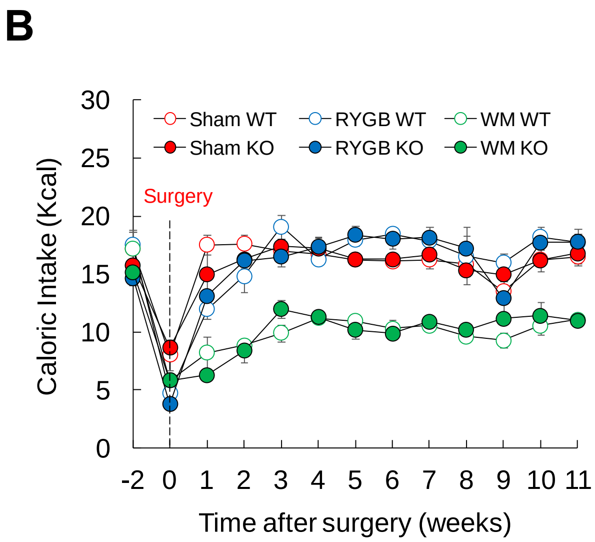

Supplemental Data Legends

Supplemental Fig. S1. Effect of RYGB on body weight and food intake in C57BL/6 mice. Male mice were placed on a two-choice diet (regular chow and 60% high-fat) for 14 weeks after which they either had undergone Sham (n=9) or RYGB surgery (n=11), or caloric restriction (n=6; weight-matched; WM) that is 35% of total intake of RYGB mice to match their body weight. Chow-fed mice without surgery (n=5) were added as an additional control group. **A)** Daily body weight and **B)** caloric intake (kcal) for 2 weeks post-surgically. Analyzed by two-way repeated measures ANOVA followed by Bonferroni post-hoc test.

Supplemental Fig. S2. RYGB improves insulin sensitivity in HF-fed obese mice. Fasting blood parameters were evaluated from the trunk blood collected at the end of sacrifice. **A)** Blood glucose by a hand-held glucometer. **B)** Serum insulin was measured by Milliplex assay. **C)** Homeostatic Model Assessment for Insulin Resistance (HOMA-IR) was calculated by fasting glucose and insulin values. Analyzed by one-way ANOVA followed by Tukey's post-hoc test. n=5-11/group; * $p<0.05$

Supplemental Fig. S3. RYGB surgery leads to a similar weight loss in FGF21 KO mice. 8-week-old male WT (n=3-4/group) or FGF21 KO mice (n=7-8/group) were fed a two-choice diet (regular chow or 60% high-fat diet) for 10 weeks before subjected to Sham or RYGB surgery or caloric restriction. **A)** Weekly body weight and **B)** caloric intake before and after surgery for 11 weeks. Analyzed by two-way repeated measures ANOVA followed by Bonferroni post-hoc test.
